# P-1974. Predicting Bacteremia in Emergency Departments: A Suite of Data-Driven Clinical Decision Tools

**DOI:** 10.1093/ofid/ofaf695.2141

**Published:** 2026-01-11

**Authors:** Nicholas P Marshall, Fatemeh Amrollahi, Fateme Nateghi Haredasht, Stephen Ma, Manoj Maddali, Amy Chang, Stan Deresinski, Niaz Banaei, Mary Kane Goldstein, Steven Asch, Jonathan H Chen

**Affiliations:** Stanford University, Palo Alto, CA; Stanford University, Palo Alto, CA; Stanford University, Palo Alto, CA; Stanford, Palo Alto, California; Stanford University, Palo Alto, CA; Stanford University, Palo Alto, CA; Stanford Health Care, Stanford, CA; Stanford University School of Medicine, Palo Alto, CA; Stanford University, Palo Alto, CA; Stanford University, Palo Alto, CA; Stanford University, Palo Alto, CA

## Abstract

**Background:**

The global blood culture bottle shortage in 2024 highlighted the critical need to optimize test utilization. While blood cultures are essential for diagnosing bacteremia, fewer than 10% yield true-positive findings. Overuse increases the risk of contamination unnecessary antibiotic exposure and hospitalizations, and strains resources. To address this, we developed a suite of predictive models leveraging structured and unstructured electronic health record (EHR) data to better stratify bacteremia risk and support targeted blood culture ordering.

**Table 1:**
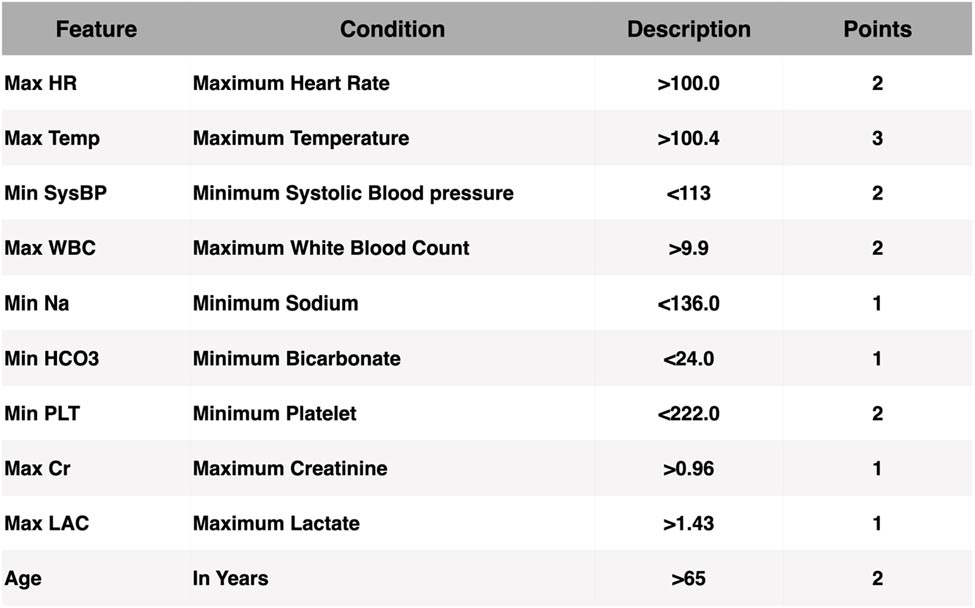
BactoScore Scoring System

This table summarizes conditions assigned to each feature in the BactoScore system, derived from the coefficients of the BactoRisk model. This design ensures straightforward application in clinical settings. A cumulative score of 4 or higher indicates a high likelihood of a positive blood culture, achieving a sensitivity of 0.95.

**Table 2:**
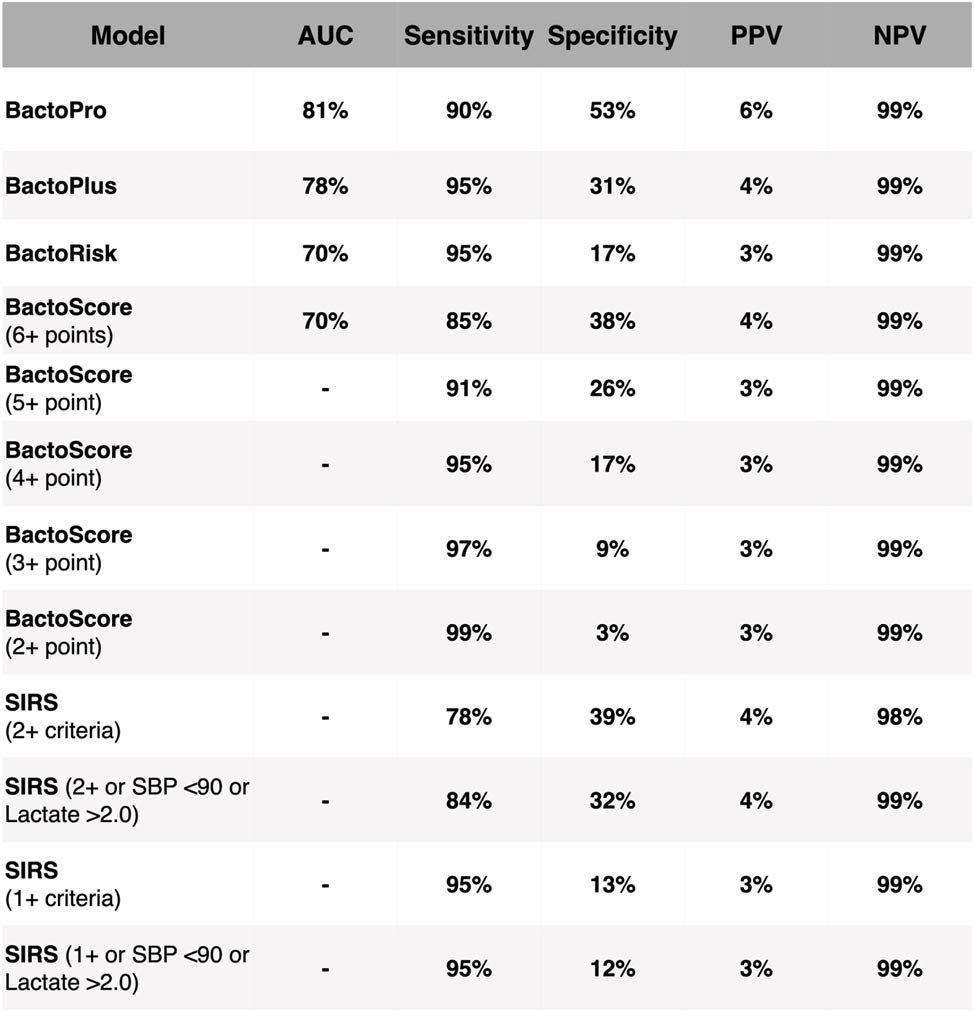
Comparison of the performance of BactoPro, BactoPlus, BactoRisk, and BactoScore against common SIRS-based criteria (Systemic Inflammatory Response Syndrome) and the numerical components of the Shapiro method

While the full Shapiro method involves a broad range of features, this comparison focuses solely on the numerical components of Shapiro. BactoPlus incorporates additional features such as prior antibiotic usage and diagnoses, which enhance the model’s predictive power. Our results demonstrate that both BactoRisk and BactoScore outperform the common SIRS-based criteria and the Shapiro method, highlighting their potential for practical use in EDs. Furthermore, the BactoScore model allows flexibility in setting the most appropriate sensitivity threshold for culture ordering based on the clinical scenario. At a sensitivity of 91%, clinicians should order the test when 5 or more criteria are met.

**Methods:**

We analyzed 135,483 ED blood cultures from patients (≥18 years, no recent bacteremia) at Stanford and Tri-Valley Hospitals (2015–2023). The primary outcome was true bacteremia (excluding contaminants). We developed three models: (1) BactoScore, a simplified, interpretable point-based tool derived from BactoRisk, a logistic regression model using structured clinical and laboratory data; (2) BactoPlus, an XGBoost-based model extending BactoRisk with diagnosis codes and recent antibiotic exposure; and (3) BactoPro, a multimodal model incorporating clinical notes via a large language model (LLM) alongside structured EHR data. Models were compared to SIRS and Shapiro’s score.

**Table 3:**
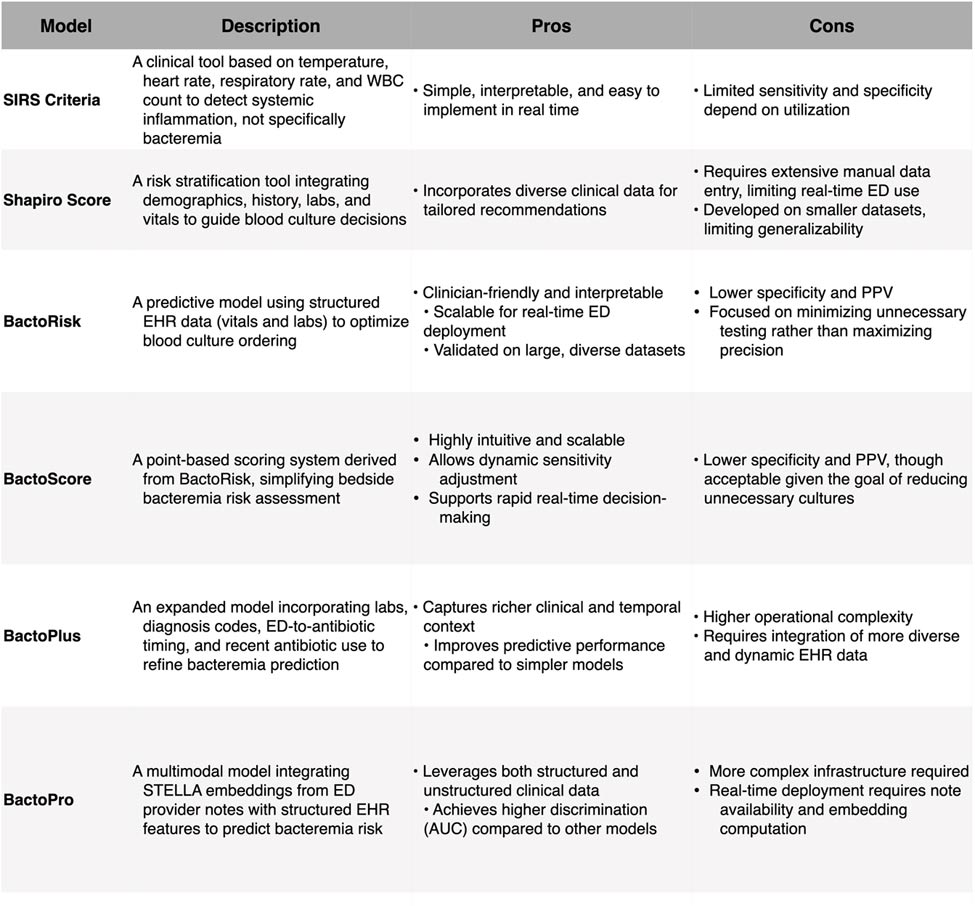
Comparison of SIRS Criteria, Shapiro, BactoRisk, BactoPlus, BactoPro, and BactoScore Models for Blood Culture Optimization This table summarizes the key features, pros, and cons of each model, highlighting their applicability, real-time implementation potential, and performance in predicting bacteremia risk and reducing unnecessary blood culture orders in ED settings.

**Results:**

At 90% sensitivity, all three models outperformed SIRS and Shapiro. BactoPro achieved the highest accuracy by combining clinical notes with structured features. BactoPlus offered comparable performance using enriched structured features. BactoScore, while simpler, retained strong predictive power and interpretability, enabling bedside use with flexible thresholds. At a 4+ point cutoff, BactoScore achieved 95% sensitivity and supported safe culture reduction with minimal missed bacteremia.

**Conclusion:**

Our tiered suite of predictive models, from the interpretable BactoScore to the advanced, multimodal BactoPro, offers flexible options for improving blood culture stewardship by accurately predicting bacteremia than existing standards. These tools support real-time risk stratification, targeted ordering, and better resource utilization in ED settings. By aligning model complexity with available infrastructure, health systems can select scalable solutions that optimize care while preserving diagnostic safety.

**Disclosures:**

Jonathan H. Chen, MD, PhD, Reaction Explorer: Ownership Interest

